# Foraging behavior of the sea urchin *Mesocentrotus nudus* exposed to conspecific alarm cues in various conditions

**DOI:** 10.1038/s41598-021-94969-w

**Published:** 2021-08-02

**Authors:** Xiaomei Chi, Mingfang Yang, Fangyuan Hu, Xiyuan Huang, Yushi Yu, Yaqing Chang, Qingzhi Wang, Chong Zhao

**Affiliations:** 1 Key Laboratory of Mariculture & Stock Enhancement in North China’s Sea, Ministry of Agriculture and Rural Affairs, Dalian Ocean University, Dalian, 116023 China; 2 Liaoning Ocean and Fisheries Science Research Institute, Dalian, 116023 China

**Keywords:** Ecology, Ecology, Ocean sciences

## Abstract

Conspecific alarm cues crushed from *Mesocentrotus nudus* prevent sea urchins from foraging the kelp, but do not repel them far away from the kelp. However, it remains largely unknown of whether this phenomenon was affected by conspecific alarm cues or by the attraction of the kelp. The present study found no significant difference in the duration in the danger area with or without the kelp around conspecific alarm cues. This suggests that the phenomenon is the strategy of sea urchins but not by the attraction of kelp. We found that conspecific alarm cues appearing between the kelp and sea urchins significantly affected foraging behavior of sea urchins fasted for 21 days. This indicates that conspecific alarm cues can effectively prevent fasted sea urchins from foraging the kelp. Further, there was no correlation between foraging velocity and the duration in the danger area. Pearson correlation analysis revealed no significant correlation between foraging velocity and the duration in the safety area close to different amounts of conspecific alarm cues, suggesting that conspecific alarm cues prevent sea urchins with strong foraging ability to forage. Collectively, the present results indicate that conspecific alarm cues as highly available biological barriers are cost-effective approaches to preventing overgrazing of sea urchins in the protection of kelp beds ecosystems. Notably, the present study is a short-term laboratory investigation that does not consider the complexity of natural conditions. Future studies are essential to test the present findings in the field.

## Introduction

Kelp beds provide food and shelters for marine organisms and thus play a key role in the marine ecosystems^1,2^. However, the transition of kelp beds to barrens occurs as a result of the overgrazing by sea urchins^3–5^. Sea urchins can search and subsequently destructively grasp the kelp. It is therefore important to establish cost-effective methods to regulate foraging behavior of sea urchins. Obvious escape behavior was observed in sea urchins exposed to the crushed conspecifics^6,7^. Further, our previous study found that conspecific alarm cues from crushed conspecific around the kelp significantly affected foraging behavior of the sea urchin *Mesocentrotus nudus* and prevented sea urchins from foraging the kelp (unpublished data). This indicates that conspecific alarm cues probably have valuable application in regulating the foraging behavior of sea urchins. However, we found that sea urchins were not far away from the kelp (unpublished data). This indicates a potential threat to the kelp beds. Thus, it is worth investigating whether this phenomenon is the strategy of sea urchins to avoid conspecific alarm cues or because of the attraction of kelps.

The sea urchin *Echinometra mathaei* fasted for 34 days increased foraging behavior^8^. This indicates starved urchins are a potential threat to the kelp beds. Our previous study suggests that conspecific alarm cues are applicative as a barrier in critical areas to prevent sea urchins from foraging the kelp (unpublished data). However, it is unclear whether this method is effective in preventing starved sea urchins, for example, from the barrens. Thus, it is essential to investigate foraging behavior of fasted sea urchins under the condition of conspecific alarm cues.

*Mesocentrotus nudus* is a representative marine herbivore, which distributes in kelp beds and barrens in China, Primorskyi Kray in Russia and Japan^9^. Destructively overgrazing kelps by sea urchins probably induce the transition of kelp beds to barrens^5^. *Mesocentrotus nudus* has obvious escape behavior in exposure to conspecific alarm cues^7^. Obviously suppressed foraging behavior was observed in *M*. *nudus* exposed to the conspecific alarm cues (unpublished data). The main purposes of the present study are to investigate (1) whether *M. nudus* are attracted by the kelp exposed to conspecific alarm cues; (2) whether foraging behavior of fasted *M. nudus* is affected when they encounter conspecific alarm cues; (3) whether foraging ability of *M. nudus* links to their responses to conspecific alarm cues.

## Results

### Experiment 1: whether *M*. *nudus* are attracted by the kelp exposed to conspecific alarm cues

**Figure 1:**
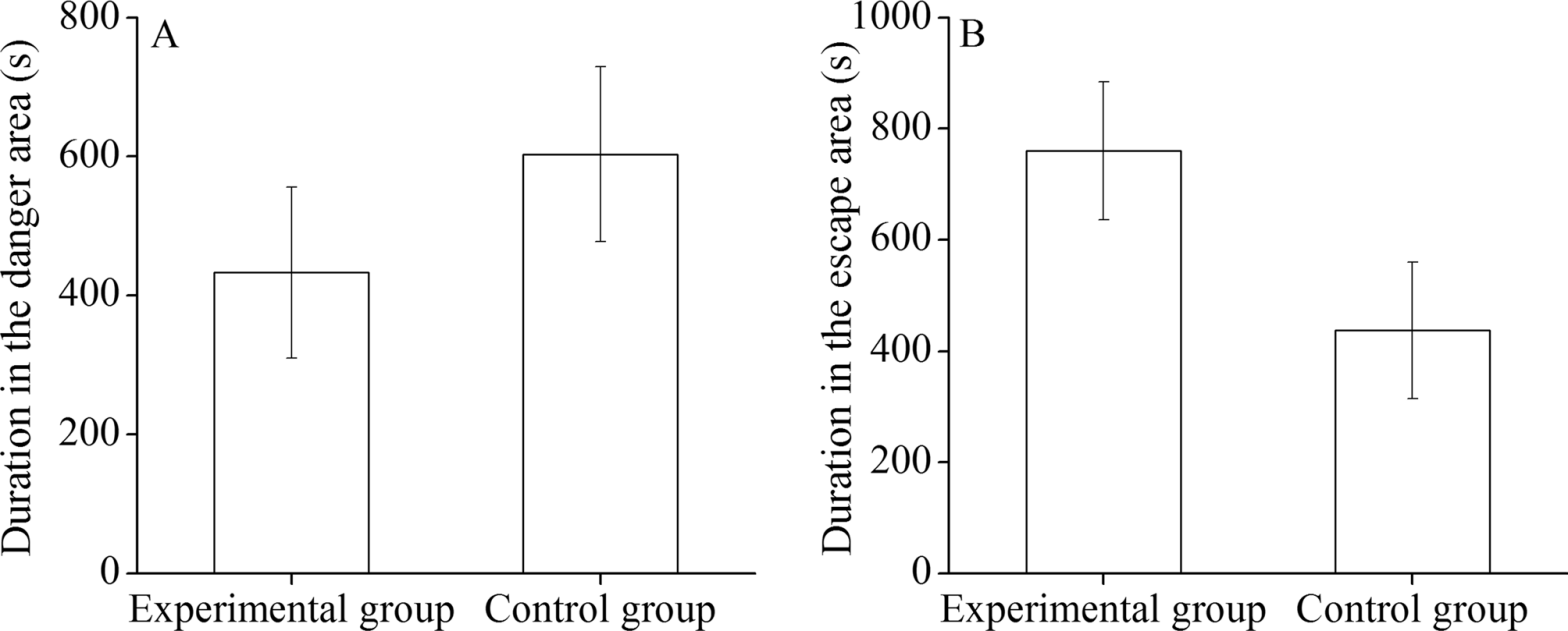
Experiment 1: duration in the danger area (**A**) and in the escape area (**B**) in the experimental and control groups.

Time spent in the danger area (b area) (*t* = − 0.966, *P* = 0.340, Fig. 1A) and the escape area (a area) (*t* = 1.854, *P* = 0.072, Fig. 1B) of sea urchins showed no significant difference between the two groups.

### Experiment 2: whether foraging behavior of fasted *M*. *nudus* is affected when they encounter the conspecific alarm cues

Five of twenty (5/20) sea urchins fasted for 7 days successfully foraged towards conspecific alarm cues, four of twenty (4/20) sea urchins fasted for 14 days successfully foraged towards conspecific alarm cues and three of twenty (3/20) sea urchins fasted for 21 days successfully foraged towards conspecific alarm cues.

**Figure 2:**
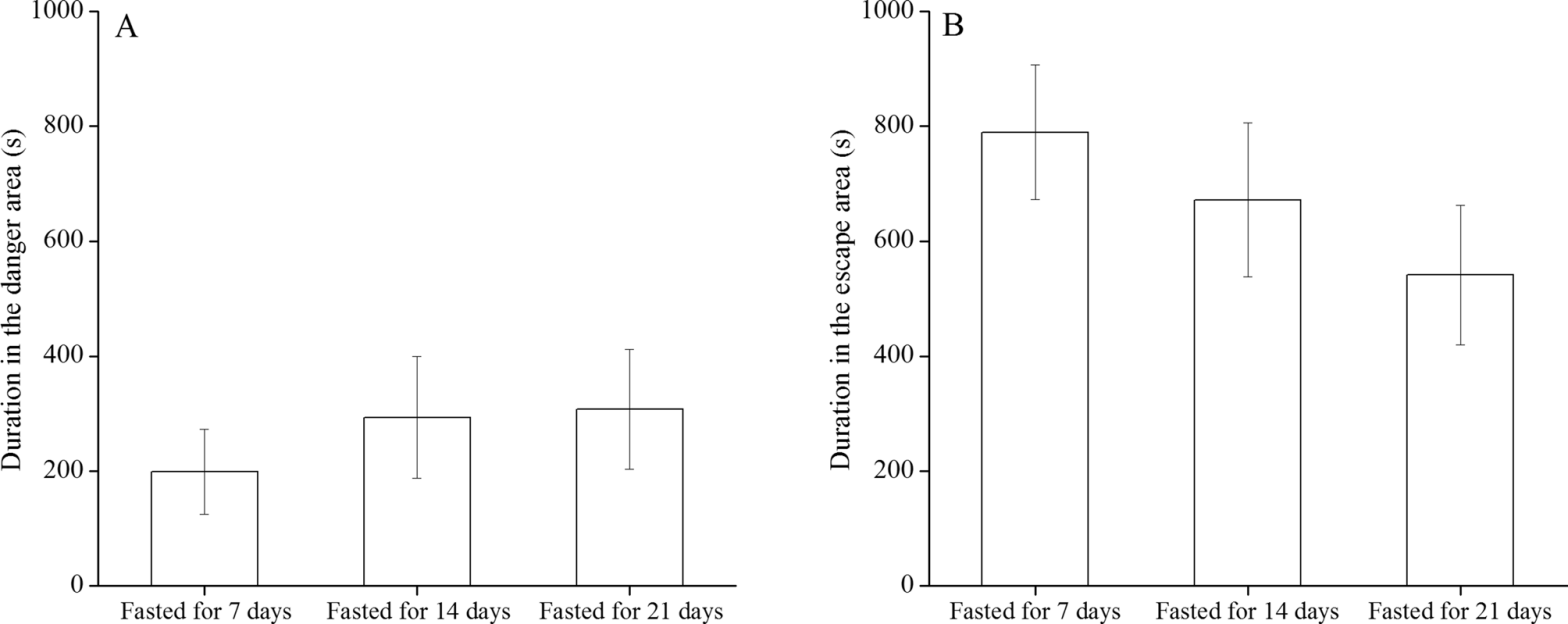
Experiment 2: duration in the danger area (**A**) and in the escape area (**B**) in *Mesocentrotus nudus* (fasted for 7, 14 and 21 days) exposed to conspecific alarm cues.

Duration in the danger area (e area) showed no significant difference between the sea urchins fasted for 7 days and the individuals fasted for 14 days (*P* = 0.489). The time in the danger area (e area) spent by sea urchins fasted for 7 days showed no significant difference to that of individuals fasted for 21 days (*P* = 0.429). Consistently, duration in the danger area (e area) showed no significant difference between sea urchins fasted for 14 days and 21 days (*P* = 0.920, Fig. 2A).

Duration in the escape area (f area) showed no significant difference between sea urchins fasted for 7 days and 14 days (*P* = 0.506). Sea urchins fasted for 7 days spent no significantly different time in the escape area (f area) to that of individuals fasted for 21 days (*P* = 0.104). Duration in the escape area (f area) showed no significant difference between sea urchins fasted for 14 days and 21 days (*P* = 0.329, Fig. 2B).

### Experiment 3: whether foraging ability of *M*. *nudus* links to their responses to conspecific alarm cues

**Figure 3:**
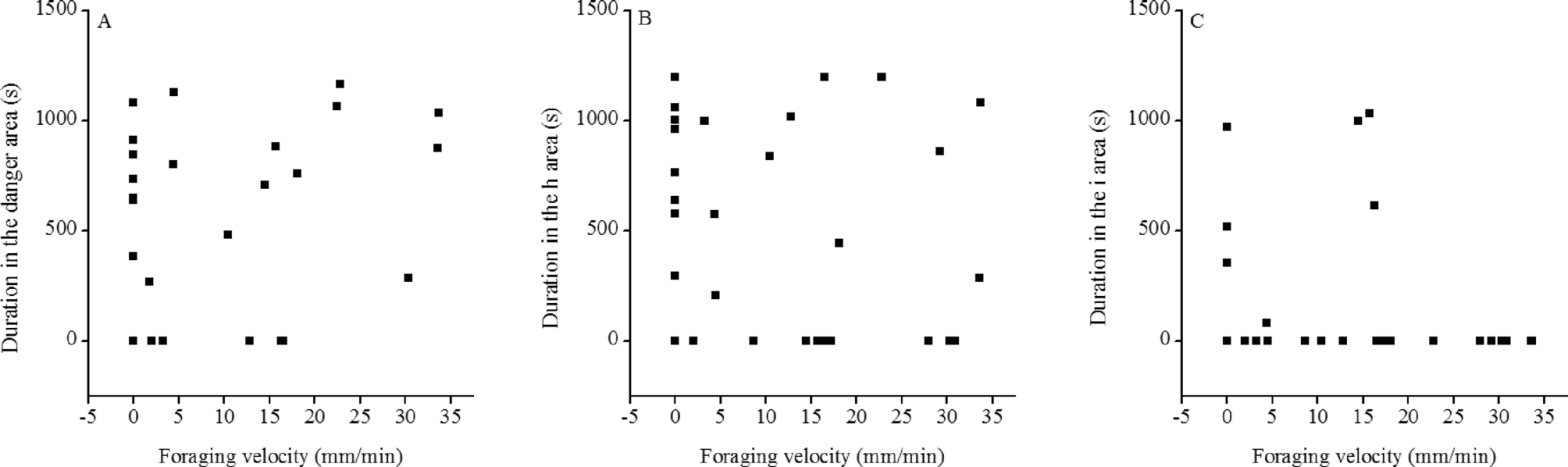
Experiment 3: correlation between foraging velocity and the duration in the danger area (g area) in *Mesocentrotus nudus* when they encountered conspecific alarm cues on the way to forage (**A**), correlation between foraging velocity and the duration in the h area (**B**), correlation between foraging velocity and the duration in the i area (**C**) when the kelp was around 5 mL and 0.5 mL conspecific alarm cues.

Two of thirsty-one (2/31) sea urchins foraged towards conspecific alarm cues when conspecific alarm cues appeared on the way to the kelp. Pearson correlation analysis revealed that the duration in the g area of sea urchins was not significantly correlated to their foraging velocity (*R*^*2*^ = 0.257, *P* = 0.195, Fig. 3A).

Only one sea urchin foraged successfully to the area of 5 mL conspecific alarm cues. No significant correlation was found between foraging velocity and the time in the h area where sea urchins spent (*R*^*2*^ = − 0.043, *P* = 0.814, Fig. 3B). There was no significant correlation between the foraging velocity and the duration in the i area of sea urchins (*R*^*2*^ = − 0.096, *P* = 0.596, Fig. 3C).

## Discussion

Sea urchins escape from conspecific alarm cues released from the injured conspecifics^7,10^. Our previous study found that sea urchins were not far away from the kelp when conspecific alarm cues were around the kelp (unpublished data). However, it remains largely unknown whether this phenomenon was affected by conspecific alarm cues or by the attraction of the kelp. The present study found no significant difference in the duration in the danger (b area) and the escape area (a area) with or without the kelp around conspecific alarm cues. This suggests that the food attraction did not affect the escape strategy induced by conspecific alarm cues. The movement distances over which sea urchins in response to predation cues range from less than one meter^10,11^ to several meters^12^. Therefore, the current result indicates adding conspecifics alarm cues around the kelp is an effectively approach to reducing the threat from sea urchins to the kelp beds.

Conspecific alarm cues that appear between the kelp and the sea urchins significantly prevent foraging behavior of sea urchins (unpublished data). However, it remains unknown of whether adding conspecific alarm cues in critical areas probably prevent fasted sea urchins to forage. Sea urchins tend to move from the barrens with scarce macroalgae to the kelp beds with plentiful macroalage^13^. Starvation significantly enhances the foraging frequency of the sea urchin *Echinometra mathaei*^8^. Thus, effectively preventing fasted sea urchins from foraging the kelp is important for the management of kelp beds. In the present study, sea urchins fasted for 21 days did not move through the area with conspecific alarm cues when conspecific alarm cues appeared between the kelp and sea urchins. Besides, duration in the escape (f area) and danger (e area) areas did not differ significantly in the sea urchins fasted for 21 days. These results suggest that escape behavior overwhelms foraging behavior in fasted sea urchins when they are exposed to conspecific alarm cues. Although the sea urchin *Heliocidaris crassispina* fasted for one week was less sensitive to the cues of dead urchins^14^, *M*. *nudus* fasted for three weeks was sensitive to the conspecific alarm cues. It is likely that conspecific alarm cues from different sea urchin species have different effects on the fasted individuals. Together with our previous study, we suggest that conspecific alarm cues can effectively prevent the fasted sea urchins (at least *M*. *nudus*) from foraging the kelp.

Increasing evidences suggest that individual differences in foraging behavior are regulated by animal personality traits^15^. Bolder threespine sticklebacks *Gasterosteus aculeatus* tend to have strong foraging behavior^16^. Sea urchins overgraze the kelp through their strong foraging tendency. Thus, we hypothesize that the sea urchin with strong foraging ability probably moves through the area of conspecific alarm cues. The present study unexpectedly found that there was no correlation between sea urchins with strong foraging velocity and the duration in the danger area (g area). There was no significant correlation between sea urchins with foraging velocity and the duration in the h area. The duration in the i area of sea urchins also showed no significant correlation with the foraging velocity. These results suggest that conspecific alarm cues as an effectively biological barrier can also prevent sea urchins (at least *M*. *nudus*) with strong foraging ability to forage.

Collectively, these results indicate that conspecific alarm cues as highly available biological barriers are a cost-effective approach to preventing overgrazing of sea urchins (at least *M*. *nudus*) in the protection of kelp bed ecosystems. Notably, the present study is a short-term laboratory investigation that does not consider the complexity of natural conditions. Future studies are essential to test the present findings in the field.

## Materials and methods

### Sea urchins

Sea urchins (~ 2 cm of test diameter) were transported from Dalian Haibao Fishery Company (121° 22′ E, 38° 77′ N) to the Key Laboratory of Mariculture and Stock Enhancement in North China’s Sea, Ministry of Agriculture and Rural Affairs (121° 56′ E, 38° 87′ N) in November 2020 and maintained in the fiberglass tank of 139 L (750 × 430 × 430 mm) of the recirculating system (Huixin Co., China) at ~ 14 °C. During the experiment period, the salinity of the recirculated seawater was 30.07–30.25‰. Sea urchins were fed the kelp *Saccharina japonica *ad libitum with aeration before the experiments. The seawater was changed every three days to remove feces and algal debris.

### Conspecific alarm cues

Conspecific alarm cues were made from crushing one *M*. *nudus* in 50 mL of fresh seawater and then filtered using a fine silk net (260 μm of mesh size)^6^. Fresh conspecific alarm cues were prepared before each behavioral experiment. Five mL of conspecific alarm cues was used to simulate the natural conditions, in which a fraction of body fluids from a sea urchin instantly released into the surrounding water^6^.

### Experiment 1: whether *M*. *nudus* are attracted by the kelp exposed to conspecific alarm cues

**Figure 4:**
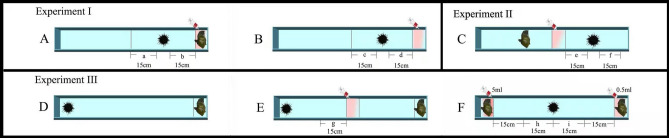
Foraging devices for the experiments with *Mesocentrotus nudus*. Experiment 1: the kelp was attracted by sea urchins (**A**,**B**); the positions of a, c and f refer to the escape areas, while positions of b, d and e refer to the danger areas. Experiment 2: five mL of conspecific alarm cues were put in the middle of the device (**C**). Experiment 3 (**D**–**F**): foraging behavior of *M*. *nudus* at 70 cm in the first trial (**D**); five mL of conspecific alarm cues were put in the middle of the device in the second trial (**E**); the position of g refers to the danger area; different amounts of conspecific alarm cues (5 mL and 0.5 mL) were put in the left and right of the devices in the third trial (**F**); the positions of h and i refer to the safety areas close to the areas with more and less conspecific alarm cues, respectively. This figure was created using the Adobe Photoshop (version CS5) software.

A piece of wild fresh *S. japonica* (~ 5 g) was placed on the side of the device with the raceways (70 × 6 × 5 cm) and 5 mL of conspecific alarm cues were subsequently added above it in the experimental group (Fig. 4A). Five mL of conspecific alarm cues were added in the control group, while the kelp was not involved (Fig. 4B). The sea urchin was individually placed 15 cm away from the kelp for each group (Fig. 4A,B). Foraging behavior of sea urchins was recorded for 20 min using a digital video recorder (FDR-AXP55, SONY, Japan). The danger area (b area) refers to the position 15 cm away from the kelp and the escape area (a area) refers to the position 15 cm away from the sea urchin (Fig. 4A,B). The time of sea urchins spent in the danger (a area) and escape (b area) areas was calculated individually for each group. The experiment was repeated 20 times using different sea urchins for each group (N = 20). The seawater was changed and the experimental device was washed for each trial to avoid potential non-experimental effects.

### Experiment 2: whether foraging behavior of fasted *M*. *nudus* is affected when they encountered conspecific alarm cues

This experiment investigated the effects of conspecific alarm cues on the foraging behavior of fasted sea urchins. The experiment group was set to simulate that conspecific alarm cues appear on the way to kelp beds (Fig. 4C). Sea urchins were fasted for 7, 14 and 21 days in the experiment groups.

An acrylic device with the raceways (70 × 6 × 5 cm) was designed according to the method of our previous study^17^ with some revisions. Five mL of conspecific alarm cues were added in the middle of the device, the wild fresh *S. japonica* (~ 10 g) was placed 15 cm away from the left side of the device, while the sea urchin was placed on 15 cm away from the right of the device (Fig. 4C). Foraging behavior of sea urchins was recorded for 20 min using a digital video recorder (FDR-AXP55, SONY, Japan). Arriving at the kelp within 20 min was defined as successfully foraging^18^. The danger area (e area) refers to the position between conspecific alarm cues and the sea urchin, while the escape area (f area) refers to the 15 cm position away from the right side of the sea urchin (Fig. 4C). The time of sea urchins spent in the e and f areas was calculated individually for all the groups. The individual experiment was repeated 20 times with different sea urchins for each group (N = 20).

### Experiment 3: whether foraging ability of *M*. *nudus* links to their responses to conspecific alarm cues

An acrylic device with the raceways (70 × 6 × 5 cm) was designed for the measurement of foraging behavior of sea urchins. Experiment 3 included three trials.

In the first trial, we measured the foraging ability of sea urchins. Ten grams of the kelp was placed on one side of the tank and one sea urchin was placed on the other side of the tank (Fig. 4D). Foraging behavior was recorded for 20 min using a digital video recorder (FDR-AXP55, SONY, Japan). Movement and velocity of sea urchins were calculated using the software ImageJ (version 1.51 n). The experiment was individually repeated 31 times using different sea urchins (N = 31). At the end of this foraging experiment, 31 sea urchins were recorded in individual cylindrical plastic cages in the tank. All the sea urchins were subsequently measured for the second and third trials (N = 31). To avoid potential fatigue influences, each behavioral experiment was carried out after another 24 h.

The second trial was carried out to test the hypothesis that sea urchins with strong foraging ability are affected when conspecific alarm cues appear on the way to the kelp. A piece of wild fresh kelp (~ 10 g) was placed on the left side of the device (70 × 6 × 5 cm), while 5 mL of conspecific alarm cues were put in the middle of the device. The individual was placed on the right of the device (Fig. 4E). Foraging behavior of sea urchins was recorded for 20 min using a digital video recorder (FDR-AXP55, SONY, Japan). The danger area (g area) refers to the 15 cm position away from conspecific alarm cues. The time of the 31 sea urchins spent in the g area was individually calculated.

We investigated whether different amounts of conspecific alarm cues around the kelp affected the sea urchins with strong foraging ability in the third trial. More conspecific alarm cues (5 mL) and a piece of wild fresh kelp (~ 10 g) were placed on the left side of the device (70 × 6 × 5 cm), while less conspecific alarm cues (0.5 mL) and wild fresh kelp (~ 10 g) were placed on the right side of the device (Fig. 4F). Sea urchins were individually placed in the middle of the device. Foraging behavior of sea urchins was recorded for 20 min using a digital video recorder (FDR-AXP55, SONY, Japan). The number of sea urchins that successfully foraged to areas with more and less conspecific alarm cues was recorded. The area h refers to the safety area that was 15 cm position away from the sea urchin, which was close to the side with more conspecific alarm cues. The area of i refers to the safety area that was 15 cm position away from the sea urchin, which was close to the side with less conspecifics alarm cues. The time spent in the h and i areas was individually calculated in 31 sea urchins.

### Statistical analysis

Normal distribution and homogeneity of variance of the data were analyzed using the Kolmogorov–Smirnov test and Levene test, respectively. Independent-sample *t*-test was carried out to compare the differences of duration in danger (b and d) and escape (a and c) areas in experiment 1. One-way ANOVA was used to analyze the duration in danger (e) and escape (f) areas of the sea urchins fasted for 7, 14 and 21 days. Pairwise multiple comparisons were carried out using LSD test when significant differences were found in the ANOVAs. Correlations between foraging velocity and duration in the g, h and i areas were analyzed using Pearson correlation analysis. A probability level of *P* < 0.05 was considered significant. All data analyses were performed using SPSS 21.0 statistical software. Graphs 2−4 were performed using Origin 9.0 software (OriginLab, USA).
